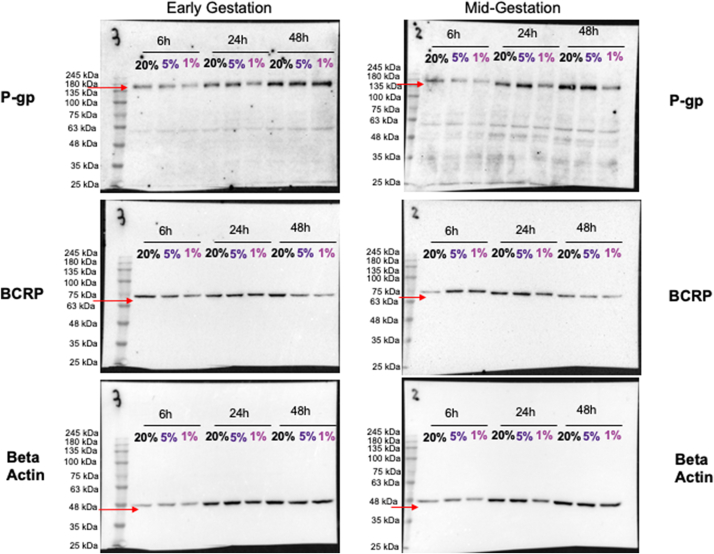# Hypoxia modulates P-glycoprotein (P-gp) and breast cancer resistance protein (BCRP) drug transporters in brain endothelial cells of the developing human blood-brain barrier

**DOI:** 10.1016/j.heliyon.2024.e30207

**Published:** 2024-04-30

**Authors:** Hafsah Mughis, Phetcharawan Lye, Guinever E. Imperio, Enrrico Bloise, Stephen G. Matthews

**Affiliations:** a Department of Physiology, Temerty Faculty of Medicine, University of Toronto, Toronto, Ontario, Canada; b Sinai Health System, Lunenfeld-Tanenbaum Research Institute, Toronto, Ontario, Canada; c Departmento de Morfologia, Universidade Federal de Minas Gerais, Belo Horizonte, Minas Gerais, Brazil; d Department of Obstetrics & Gynaecology, Temerty Faculty of Medicine, University of Toronto, Toronto, Canada; e Department of Medicine, Temerty Faculty of Medicine, University of Toronto, Toronto, Ontario, Canada

**Keywords:** Blood-brain barrier (BBB), Brain development, Hypoxia, Multidrug resistance (MDR) transporters, P-glycoprotein (P-gp/*ABCB1*), Breast cancer resistance protein (BCRP/*ABCG2*), Oxygen

## Abstract

P-glycoprotein (P-gp) and Breast Cancer Resistance Protein (BCRP) multidrug resistance (MDR) transporters are localized at the luminal surface of the blood-brain barrier (BBB). They confer fetal brain protection against harmful compounds that may be circulating in the peripheral blood. The fetus develops in low oxygen levels; however, some obstetric pathologies such as pre-eclampsia, placenta accreta/previa may result in even greater fetal hypoxic states. We investigated how hypoxia impacts MDR transporters in human fetal brain endothelial cells (hfBECs) derived from early and mid-stages of pregnancy. Hypoxia decreased BCRP protein and activity in hfBECs derived in early pregnancy. In contrast, in hfBECs derived in mid-pregnancy there was an increase in P-gp and BCRP activity following hypoxia. Results suggest a hypoxia-induced reduction in fetal brain protection in early pregnancy, but a potential increase in transporter-mediated protection at the BBB during mid-gestation. This would modify accumulation of various key physiological and pharmacological substrates of P-gp and BCRP in the developing fetal brain and potentially contribute to the pathogenesis of neurodevelopmental disorders commonly associated with *in utero* hypoxia.

## 1 Introduction

The developing blood-brain barrier (BBB) represents the primary barrier regulating transfer of key physiological substrates in and out of the brain and protects the developing brain against drugs and toxins present in the fetal circulation. Brain endothelial cells (BECs) form brain microvessels and are surrounded by pericytes and astrocytes [1,2]. BECs express tight junction proteins and the multidrug resistance (MDR) transporters, P-glycoprotein (P-gp, encoded by *ABCB1*) and breast cancer resistance protein (BCRP encoded by *ABCG2*) [3]. The MDR transporters decrease entry of neurotoxins and drugs, as well as endogenous compounds such as cytokines, steroids and hormones into the brain extracellular space, supporting brain development and providing brain protection [2,[4], [5], [6]].

Expression and function of P-gp and BCRP is developmentally regulated at the BBB. Their expression has been detected within human cerebral microvessels between 5-8-weeks of gestation [7]. Recent studies have demonstrated that these transporters are functional in human fetal BECs (hfBECs) from early gestation [8]. Their protective role is essential because approximately 70 % of pregnant women are prescribed drugs [9], many of which are P-gp and BCRP substrates [10,11]. However, limited information is available as to the effects of pregnancy complications on these transporters in the developing BBB, especially those involving fetal hypoxia.

Oxygen levels in the embryo and fetus are very low in the first trimester but increase as pregnancy progresses. Overall, oxygen concentrations in the embryo range between 2 and 9 %, *in vivo* [12]. In the placenta, levels range between 2.5 and 3.3 % before 10 weeks of pregnancy, rising to approximately 8 % at 14 weeks [13,14]. Placental oxygen levels decline to approximately 6 % near the end of pregnancy, due to rapid fetal growth, which increases oxygen demands from the placenta [13]. In normal pregnancies, oxygen concentrations at term are ∼3.7 % and ∼2.5 % in the umbilical vein and artery, respectively [13], and the fetus regulates oxygen in such a way that supply exceeds its metabolic demands. However, instances of oxygen deprivation or fetal hypoxia can lead to severe damage to vital organs, especially in the developing brain [15,16]. Hypoxia can occur in numerous situations in pregnancy including preeclampsia (PE), placenta accreta/previa, placental insufficiency and cardiovascular disease, as well as exposure to high altitude environments, and tobacco consumption [17]. Hypoxia can modify BBB integrity leading to disruption of brain protection. Hypoxia also modifies the fetal brain epigenome, and this can lead to altered neurodevelopment and impact brain function in later life [18]. Therefore, it is critical to understand the effects of hypoxia on the microvasculature in the developing brain.

Little is known as to the effects of hypoxia on P-gp and BCRP at the BBB, and most studies have been conducted in the adult BBB in rat and mouse models [[19], [20], [21], [22], [23], [24], [25], [26]]. No studies have reported how hypoxia affects P-gp and BCRP, and therefore fetal neuroprotection, in the human developing brain microvasculature. Different cultured BEC models have been established, using guinea pig [[27], [28], [29]], rat [[30], [31], [32]], mouse [3,33,34] and human 3D BBB models [[35], [36], [37]]. However, these models are somewhat limited in terms of their physiological relevance towards the biology of hfBECs. In addition, oxygen concentrations used in these hypoxic cellular systems are in the range of 3 %–10 %, which may not represent significant hypoxia for the developing brain [38]. It has been reported that optimum (physiological) oxygen concentration for normal development of mammalian embryos is around 5 % oxygen [39], and in most tissues during development, hypoxic responses occur around 0.5 %–1 % oxygen [38].

We hypothesized that hypoxia will modulate the expression and activity of P-gp and BCRP in primary hfBECs derived in early and mid-pregnancy. We investigated the acute and chronic effects of hypoxia on P-gp and BCRP *in vitro,* in human primary hfBECs isolated in early (∼12 weeks) and mid (∼18 weeks) pregnancy.

## 2 Methods

### 2.1 Cell culture and reagents

hfBECs were isolated from early and mid-gestation fetal brains as described previously [8,40,41]. Following elective termination, fetal brains were collected in early gestation and mid-gestation. All tissues were collected by the Research Centre for Women's and Infants' Health BioBank program at the Sinai Health System. Written informed consent (Protocol #18-0057-E) was acquired using a protocol approved by the Research Ethics Boards (REB) of Sinai Health and the University of Toronto. We were unable to report on any donor clinical information due to REB policies not allowing any identifying or clinical information to be collected from elective pregnancy terminations.

hfBECs were cultured at 37°C / 5 % CO_2_ in EndoGROTM-MV Complete Culture Media Kit®, (EndoGRO, SCME004, Millipore, ON, Canada) at 20 % oxygen (5 % CO_2_, 37 °C), supplemented with reagents as described previously [8,40,41]. Following isolation, hfBECs were collected and stored in liquid nitrogen. All experiments were carried out with cells in passage 4 [40]. We have previously shown no differences in the expression of both P-gp and BCRP between passage 1 and passage 4 [8].

### 2.2 Hypoxia induction in human fetal brain endothelial cells (hfBECs)

Primary hfBECs derived in early gestation (11.3–12.5 weeks; *N =*6) and mid-gestation (17.2–18.5 weeks; *N =*6) were seeded in either 6-well plates (25000 cells/cm^2^) for gene and protein analysis, or in 96-well plates (8000 cells/well) for activity assays. Cells were grown to confluence and cultured for 24-h in EndoGRO Media at 20 % oxygen (5 % CO_2_, 37 °C). After 24-h in culture, cells were exposed to 3 different oxygen tensions: 20 % (hyperoxia; control group), 5 % (physiological fetal hypoxia) or 1 % (fetal hypoxia) oxygen. Modular Incubator Chambers (MIC-101, Billups-Rothenberg, Inc., USA) were used to achieve the respective hypoxic conditions. Three chambers were used for 20 %, 5 % and 1 % oxygen treatments, and in each experiment, the respective chamber was flushed with 5 % oxygen (5 % oxygen, 5 % CO_2_ and N_2_ balance) and 1 % oxygen (1 % oxygen, 5 % CO_2_ and N_2_ balance) (Linde Canada), whenever opened. 24-h prior to the experiment, media was equilibrated with 1 % and 5 % oxygen [41]. For functional assays, culture media was removed and replaced with a combination of Dulbecco's Modified Eagle Medium (DMEM, 21063029, Thermo Fisher Scientific) and 10 % charcoal-stripped Fetal Bovine Serum (CS-FBS, Wisent, QC, CA) overnight, before the start of the experiment. At 0-h, the plates were transferred to modular incubator chambers, which were infused with 1 % and 5 % oxygen (5 min). Once flushed, the chambers were clamped and moved to the incubator (37 °C). For cells cultured at 20 % oxygen (control group), chamber clamps were kept open. To confirm hfBECs' response to hypoxia, vascular endothelial growth factor *(VEGF)* mRNA expression (a marker of hypoxia) was analyzed [42]. To measure oxygen levels inside the chamber, an optical OXYLogger oxygen sensor (from PreSens; Regensburg, Germany) was placed inside the 1 % chamber. Oxygen levels were monitored during hypoxia exposure to ensure no leakage of the chamber. All assays were performed at 6-, 24-, and 48-h; and analyzed to observe the acute- and longer-term effects of hypoxia on P-gp/*ABCB1* and BCRP/*ABCG2* function and expression in the developing hfBECs.

### 2.3 P-gp, BCRP and esterase activity assays

P*-*gp function was evaluated as described previously [8,40,43]. hfBECs (*N* = 6/group) were seeded and exposed to the 3 different oxygen tensions (20 %, 5 % and 1 % oxygen) as detailed above. Each subject/treatment was set up in technical triplicates on the plate. At 0-h, media was removed, and cells were washed (2x) with a mixture of warm (37 °C) Tyrode salts solution (Sigma, #T2145) and sodium bicarbonate (1 g/L, Sigma, #S6014). Cells were incubated with calcein-acetoxymethyl ester (Ca-AM), a P-gp substrate (product number 177831, 10^−6^ M, Sigma) for 1 h at 37 °C and 5 % CO_2_. Once substrate was added, the chambers containing the plates were flushed with 5 % and 1 % oxygen for 5 min and sealed. Following the 1-h incubation, accumulation of Ca-AM was measured by first placing the plates on ice and washing the cells (2x) with ice-cold Tyrode salts solution. Cells were then lysed in 200 μL cold 1 % Triton X-100 (Sigma #X100) in HBSS. A Microplate Reader was used to measure Ca-AM accumulation at excitation/emission wavelengths of 485/510 nm.

Esterase activity was measured as we have described previously [8,40,43]. Ca-AM is a non-fluorescent substrate of P-gp that is cleaved by endogenous esterases upon entering the cells, resulting in conversion to fluorescent Calcein (which is not transported by P-gp). However, cells expressing P-gp extrude Ca-AM before it can be converted to fluorescent Calcein [44]. To confirm hypoxia does not affect esterase activity, hfBECs isolated in mid-gestation (*N* = 6) were exposed to hypoxia (1 % oxygen; 6-, 24-, 48-h). After treatment, warm Tyrode salts solution was used to wash the cells which were then incubated with 10^−6^ Ca-AM in 200uL warm Lysis buffer (1 % Triton X-100 (Sigma #X100) in HBSS) for 1-h under 1 % oxygen. Using a Microplate Reader, conversion of Ca-AM to fluorescent Calcein, after treatment, was evaluated at excitation/emission wavelengths of 485/510 nm.

BCRP activity assays were carried out as described previously [8,40,43]. Briefly, hfBECs were incubated with 2 μM chlorin e6 (Ce6, Santa Cruz Biotechnology, product #SC-263067; 37 °C, 5 % CO2, 1 h). Ce6 is a photosensitizer that was identified as a specific BCRP substrate [45,46]. In the absence of any inhibitors, Ce6 is effluxed by BCRP [47]. Following incubation, plates were cooled on ice and cells rinsed once with ice-cold Tyrode salts solution. Cells were then lysed in 200 μL cold 1 % Triton X-100 (Sigma #X100) in HBSS. The Microplate Reader measured the accumulation of Ce6 at excitation/emission wavelengths of 407/667 nm.

To validate the specificity of substrates for their respective transporters, hfBECs were treated with inhibitors of P-gp (verapamil (VPL), 100 μM; sigma, product #V4629), and BCRP (Ko143, 10 μM; sigma, product #K2144) [8]. Inhibitor controls were included each time functional assays were performed.

### 2.4 Protein analysis

Cells were harvested from 6-well plates in lysis buffer (1 mol/L Tris-HCL pH 6.8, 2 % SDS, and 10 % glycerol) for protein analysis after 6-, 24-, and 48-h of treatment. The lysis buffer included a mixture of phosphatase inhibitor cocktail (78420, Thermo Scientific) and protease. Protein was then extracted by sonication. Protein was quantified using a Pierce BCA protein assay kit (Thermo Scientific). Total protein (22 μg) was loaded on 8 % SDS polyacrylamide gels at 100V for 1.5-h for electrophoretic separation. Proteins were then transferred (10 min) from gels to polyvinylidene difluoride membranes (PVDF) using the Bio-Rad Trans-Blot® Turbo™ Transfer System. Membranes were blocked for 1-h at room temperature with 5 % skim milk in Tris-Buffered Saline containing 0.1 % Tween (TBS-T). Following blocking, membranes were incubated (4 °C) with primary antibodies: P-gp (Abcam #ab170903, dilution 1:1000) and BCRP (Abcam #ab108312, dilution 1:1000, RRID: AB_10861951), for 40-h; and the loading control β-actin (Santa Cruz Biotechnology, product #SC-47778, dilution 1:2000, RRID: AB_626632) for 12-h. Membranes were then washed (3x) with TBS-T and incubated for 1-h at room temperature with HRP-linked anti-rabbit secondary antibody (1:10,000; GE Healthcare Bioscience, QC, Canada). For all early-pregnancy samples, Luminata™ Crescendo Western HRP Substrate (Millipore) was used to assess chemiluminescence. The substrate SuperSignal™ West Femto (Thermo Scientific) was used for mid-gestation P-gp and BCRP samples. Membranes were probed with their respective substrates for 5 min and detected under UV using the Bio-Rad ChemiDoc™ MP Imaging system (RRID:SCR_019037). The Image Lab™ software (RRID:SCR_014210) was used to quantify P-gp and BCRP protein bands by densitometric analysis. The bands were normalized to β-actin signal for total protein assessment.

### 2.5 Quantitative real time PCR (qPCR)

Total RNA was extracted using the RNeasy Mini Kit (Catalogue #73404, Qiagen, Canada), as described previously [8,40]. NanoDrop 1000 Spectrophotometer (Thermo Scientific) was used to determine the RNA purity/concentration (A260/A280 ratio of approximately 2.0), and total RNA (1 μg) was reverse transcribed to cDNA by means of the Bio-Rad iScriptTM Reverse Transcription Supermix. SYBR Green reagent (Sigma-Aldrich) was utilized for performing the qPCRs using the Bio-Rad CFX 380 Real-Time system C1000 TM Thermal Cycler. The following conditions were used: 1 cycle of 95 °C for 2 min, and 40 cycles of 95 °C for 5s and 60 °C for 20s. 5 ng of RNA was used per reaction and each sample was set up in technical triplicates, where the cycle threshold (CT) value of each replicate per sample was recorded. Measured genes and primer sequences, with their references, are available in Table 1. The primers were purchased from Integrated DNA Technologies (USA). The geometric mean of the reference genes succinate dehydrogenase complex flavoprotein subunit A (*SDHA*) and cytochrome C1 (*CYC1*) was calculated and used to normalize target gene values. The 2^−ΔΔCT^ Method [48] was used to calculate the relative mRNA expression of *VEGF*, *ABCB1* (encodes for P-gp) and *ABCG2* (encodes for BCRP).

**Table 1:** List of primers for qRT-PCR used in this study.

Primer name	Sequence	References
*ABCB1*	Forward: 5′- GCC CTT GTT AGA CAG CCT CA -3′	[49]
	Reverse: 5′- GGC TTT GTC CAG GGC TTC TT -3′	
*ABCG2*	Forward: 5′- TGG AAT CCA GAA CAG AGC TGG GGT -3′	[49]
	Reverse: 5′- AGA GTT CCA CGG CTG AAA CAC TGC -3′	
*VEGF*	Forward: 5′- CGG GCC TCC GAA ACC ATG AAC TT -3′	[50]
	Reverse: 5′- CCC TCC TCC TTC TGC CAT GGG T -3′	
*SDHA*	Forward: 5′- TGG GAA CAA GAG GGC ATC TG -3′	[51]
	Reverse: 5′- CCA CCA CTG CAT CAA ATT CAT G -3′	
*CYC1*	Forward: 5′- CAG ATA GCC AAG GAT GTG TG -3′	[52]
	Reverse: 5′- CAT CAT CAA CAT CTT GAC CC -3′	

### 2.6 Cell viability

Cell viability was measured using 6-well plates at 25000 cells/cm^2^ for each oxygen group (20 %, 5 % and 1 % oxygen) at each time-point (6-, 24-, and 48-h), as described above. Following treatment, cells were washed with calcium-free HBSS, incubated with Accutase (1 mL; 5 min, 37 °C, 5 % CO_2_) and then mixed with cell media (2 mL) to stop the reaction. An aliquot (100uL) of each well was mixed with equal parts of trypan blue (100uL; 5 min) (15250-061; Gibco Life Technologies), mounted onto a hemocytometer (Hausser Scientific, Pennsylvania, United States) and cells were counted under the microscope. Cells which stained blue were deemed dead and those that extruded the dye, alive. Percent viable cells was determined using the following equation: % viable cells = viable cells/(viable cells + dead cells) x 100 [8].

### 2.7 Statistical analyses

Statistical analyses were performed using Prism 9 (GraphPad Software, Inc., USA). Outliers were detected using the Grubbs' test and normality was assessed using Shapiro-Wilk test. Two-way ANOVA was utilized to analyze gene and protein expression followed by Dunnett's multiple comparisons test. This allowed comparison between the various oxygen levels at each time-point in a single analysis. For P-gp and BCRP functional assays, the one-way ANOVA was used followed by Dunnett's multiple comparisons test because data for each time-point was analyzed separately. Data are presented as mean ± standard error of the mean (S.E.M.). P < 0.05 was considered significant.

## 3 Results

### 3.1 Hypoxia upregulates *VEGF* in developing hfBECs

*VEGF* was used as a marker of hypoxia [42]. *VEGF* transcript levels were elevated under 1 % hypoxia at 24- (P < 0.05) and 48-h (P < 0.01) in early pregnancy hfBECs (Fig. 1A). In mid-pregnancy, *VEGF* transcript levels were significantly higher in the 1 % oxygen group, compared to 20 % oxygen, at 6-h (P < 0.001), 24-h (P < 0.01), and 48-h (P < 0.05) (Fig. 1B). The oxygen sensor OXYLogger (Supplementary Figs. 1A–B) confirmed that oxygen levels were maintained throughout the treatment periods. Viability of hfBECs ***was not altered in any treatment group*** (Supplementary Figs. 1C–D).

**Fig. 1:**
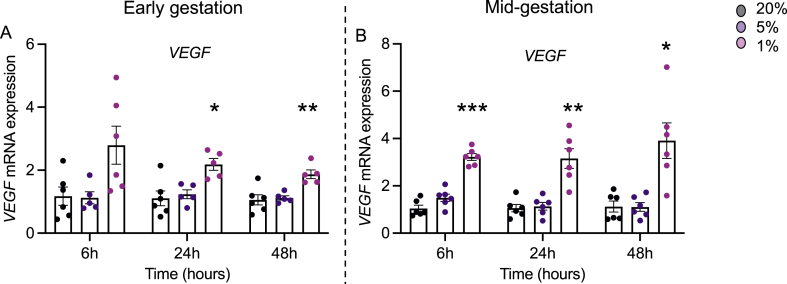
**Hypoxia upregulates *VEGF* mRNA expression in hfBECs derived from early and mid-pregnancy.** Relative *VEGF* mRNA levels in hfBECs from early **(A)** and mid- **(B)** gestation following 6-, 24-, and 48-h of hypoxia (N = 6/group). Statistical analysis: Two-way ANOVA followed by Dunnett's multiple comparisons test. Values are displayed as mean ± S.E.M. Significant difference from 20 % oxygen was set at (*) P < 0.05; (**) P < 0.01; and (***) P < 0.001.

### 3.2 Hypoxia modifies P-gp and BCRP function in a gestational-age-specific manner

In hfBECs derived from early pregnancy, we found no significant effect of 1 % and 5 % oxygen after 6-, 24- and 48-h on P-gp activity relative to 20 % oxygen, however, there was a trend (P < 0.08) for elevated P-gp activity under 1 % oxygen at 48-h (Fig. 2A–C). A significant increase in P-gp activity was detected in cells derived in mid-gestation following exposure to 1 % oxygen compared to 20 % oxygen at 6-h (P < 0.01, Fig. 2D), 24-h (P < 0.05, Fig. 2E), and 48-h (P < 0.01, Fig. 2F). Interestingly, longer-term exposure (48-h) to 5 % oxygen also led to an elevation (P < 0.05) in P-gp activity (Fig. 2F).

**Fig. 2:**
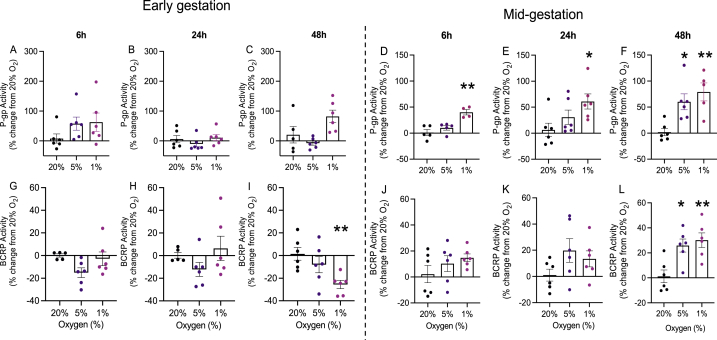
**Hypoxia decreased BCRP activity in early gestation, and increased P-gp and BCRP activity in hfBECs derived in mid-gestation.** Change in P-gp activity following hypoxia after **(A,D)** 6-h, **(B,E)** 24-h, and **(C,F)** 48-h, and BCRP activity after **(G,J)** 6-h, **(H,K)** 24-h, and **(I,L)** 48-h in hfBECs (*N =*4–6/group, if *N* < 6, an outlier has been removed). Activity is displayed as change relative to cells under 20 % oxygen (control cells represented by a solid line at zero). Statistical analysis: One-way ANOVA followed by Dunnett's multiple comparisons test. Values are displayed as mean ± S.E.M. Significant difference from 20 % oxygen was set at (*) P < 0.05; and (**) P < 0.01.

BCRP activity was also affected by hypoxia in hfBECs. In early pregnancy, there was a significant decrease in BCRP activity after 1 % oxygen exposure compared to 20 % oxygen, at 48-h (P < 0.01, Fig. 2I); no differences were detected at other time points (Fig. 2G and H). In contrast, in hfBECs derived in mid-gestation, we detected significantly higher BCRP activity following 1 % oxygen (P < 0.01) and 5 % oxygen (P < 0.05) treatment compared to 20 % oxygen, at 48-h (Fig. 2L). We detected no changes in BCRP activity after exposure to 1 % and 5 % oxygen at 6-h and 24-h (Fig. 2J and K). Of importance, we also treated the developing hfBECs with specific P-gp (verapamil - VPL) and BCRP (Ko143) inhibitors to validate our functional assays. As expected, VPL increased calcein-AM accumulation and Ko143 increased Ce6 accumulation in hfBECs derived from early and mid-pregnancy, in all oxygen treatments and time points investigated (Supplementary Fig. 2A-L). Since the P-gp activity assay depends on the actions of cellular esterase [43,44], it was important to determine that hypoxia does not impact cellular esterase activity. We observed no changes in esterase activity following 6-, 24-, and 48-h of hypoxia (1 % oxygen) in mid-gestation hfBECs (Supplementary Fig. 3).

### 3.3 Hypoxia has specific effects modulating P-gp and BCRP protein/mRNA levels

We evaluated whether oxygen tension modulates P-gp/*ABCB1* and BCRP/*ABCG2* protein and mRNA in developing hfBECs. Hypoxia had no effect on P-gp protein levels at any of the time-points investigated in hfBECs derived in early or mid-gestation (Fig. 3A–D). *ABCB1* mRNA levels did not change in early hfBECs (Fig. 4A) but were significantly decreased in mid-gestation hfBECs after exposure to 1 % oxygen (P < 0.05), compared to 20 % oxygen at 24- and 48-h (Fig. 4B). BCRP protein levels were significantly decreased (P < 0.05) after longer-term (48-h) severe hypoxia (1 % oxygen) compared to 20 % oxygen in early-gestation hfBECs (Fig. 3A &E) but did not change in hfBECs derived in mid-gestation (Fig. 3B and F). We detected no differences in *ABCG2* mRNA levels following hypoxia in either age-group (Fig. 4C and D).

**Fig. 3:**
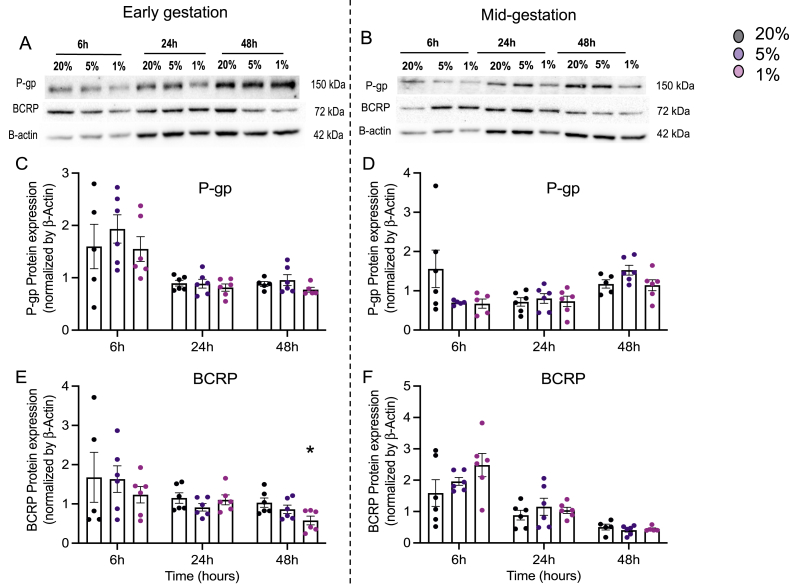
**Hypoxia decreased BCRP protein levels in hfBECs derived in early gestation.** (**A-B**) Representative Western blots of P-gp and BCRP, (**C–F**) densitometric analysis of P-gp and BCRP, normalized to β-actin, following 6-, 24-, and 48-h of hypoxia (*N =*5–6/group, if *N* < 6, an outlier has been removed). Statistical analysis: Two-way ANOVA followed by Dunnett's multiple comparisons test. Values are displayed as mean ± S.E.M. Significant difference from 20 % oxygen was set at (*) P < 0.05. Western blot membranes of P-gp and BCRP protein in **(A**–**B)** are shown in Supplementary Fig. 4.

**Fig. 4:**
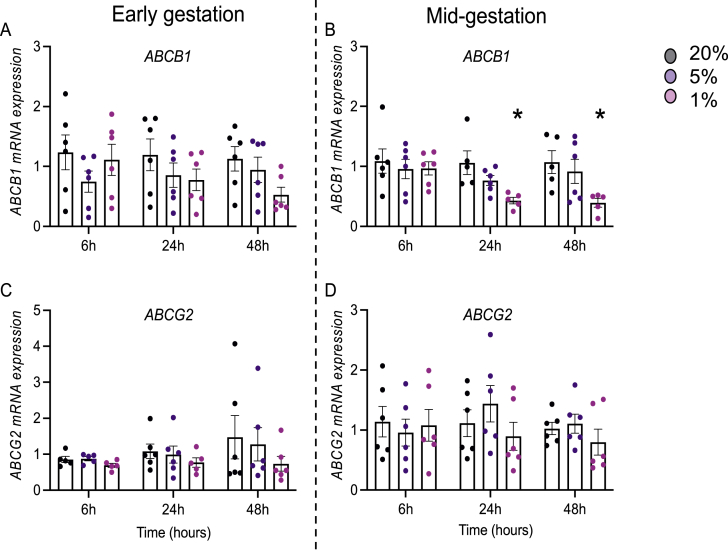
**Effect of hypoxia on *ABCB1* and *ABCG2* mRNA levels in hfBECs derived from early and mid-gestation**. Relative *ABCB1/ABCG2* mRNA in early **(A and C)** and mid- **(B and D)** gestation after 6-, 24-, and 48-h of hypoxia (*N =*5–6/group, if *N* < 6, an outlier has been removed). Statistical analysis: Two-way ANOVA followed by Dunnett's multiple comparisons test. Values are displayed as mean ± S.E.M. Significant difference from 20 % oxygen was set at (*) P < 0.05.

## 4 Discussion

In the present study, we have shown that hypoxia affects P-gp and BCRP expression and function in hfBECs derived in early and mid-pregnancy. This follows careful characterization of the phenotype of the hfBECs used in this study [8]. The expression and localization of BEC markers such as Von Willebrand factor (vWF), claudin (CLDN)5 and zonula occludens (ZO)-1 has been determined previously in these cells, as well as the functional expression of P-gp and BCRP and their ability to form tube-like structures [8].

Previous studies investigating the effects of hypoxia on P-gp and BCRP in BECs have yielded varied results, depending on the disease model, the type of hypoxic treatment and the species investigated [[19], [20], [21], [22], [23],^25^,^26^,^30^,[53], [54], [55]]. An *in vitro* study using bovine BECs showed downregulation of *ABCB1* mRNA after 4-h of concomitant glucose and oxygen deprivation [22]. Conversely, an increase in P-gp protein was detected in primary cultures of adult rat BECs following 6-h of hypoxia induced by hydrogen peroxide exposure [30]. In another study, middle cerebral artery occlusion in the mouse resulted in elevated P-gp expression and function [55]. At the level of BCRP, protein levels were decreased following sustained hypoxia (1 % oxygen) in the human BEC cell line (HBEC-5i) [25]. In contrast, there was a significant upregulation of *ABCG2* and BCRP protein levels 3–14 days after induction in a rat *in vivo* stroke model [54].

P-gp protein expression and function were not affected by hypoxia in hfBECs derived in early gestation, suggesting that fetal brain protection provided by P-gp at the developing BBB is likely maintained during hypoxic insults in early pregnancy. In contrast, in hfBECs derived in early gestation, hypoxia induced a consistent reduction in BCRP protein and activity, indicating that severe hypoxia may lead to an elevated fetal brain exposure to BCRP substrates in hypoxia-related pregnancy complications. These include xenobiotics/toxins that may be harmful for the fetus, as well as endogenous factors such as folate and sphingolipids, that are important for normal neurodevelopment [4].

The effects of hypoxia on transporter activity were more pronounced in mid-gestation hfBECs. Acute and longer-term hypoxia increased P-gp function. As such, it is possible that fetal hypoxia may result in an increase in P-gp activity decreasing the transfer of various substrates across the BBB and conferring greater protection of the developing brain. However, at the same time, there would be a decrease in the brain accumulation of physiological substrates important for normal brain development. In this connection, interleukin-6 (IL-6) is a P-gp substrate that is important in neurodevelopment. IL-6 is known to improve survival of neurons in culture, protect neurons from excitotoxic and ischemic insults, and regulate the development of astrocytes [56]. Endogenous glucocorticoids including cortisol and corticosterone are also P-gp substrates. Glucocorticoids are important for normal brain development; however, fetal glucocorticoid levels are maintained at low levels as the developing brain is extremely vulnerable to glucocorticoid excess [33]. Fetal hypoxia triggered by lower maternal oxygen or by impaired placental blood flow has been shown to increase cortisol levels in fetal circulation [57]. An increase in P-gp at the BBB following hypoxia in mid-gestation may protect the fetal brain from the effects of glucocorticoid excess.

BCRP function was also increased by severe hypoxia in mid-gestation hfBECs, but only following extended exposure. An increase in BCRP will increase fetal brain protection against entry of drugs and environmental toxins. However, like P-gp, BCRP transports a number of endogenous molecules including folic acid. Folate is particularly important during rapid tissue growth and development, and hence it is of major importance during morphogenesis and organogenesis. Folate deficiency during pregnancy can result in unfavorable outcomes including neural tube defects and preterm delivery (PTD) [58]. Thus, specific endogenous BCRP substrates are key during brain organogenesis. It is unclear how an increase in BCRP activity following severe and chronic hypoxia might impact transfer of these important endogenous factors into the fetal brain, and further studies are required to investigate this.

Overall, effects of hypoxia observed on P-gp and BCRP were different in early gestation compared to mid-gestation. P-gp activity did not change under hypoxia in early gestation but increased in mid-gestation hfBECs. However, BCRP activity significantly decreased in early gestation but increased in mid-gestation. In this regard, BECs in early and mid-gestation function in very different contexts. In early gestation, there is rapid angiogenesis and neurogenesis occurring in a relatively low oxygen environment, as the placenta is still developing [6,[59], [60], [61]]. In mid-gestation, BECs exist in a higher oxygen environment where there is rapid growth of the cerebral hemispheres and contraction of the ventricular system [41,62]. We demonstrated that BECs preserve their developmental phenotype *in vitro* [8,27,28], and this likely relates to the different transporter responses observed in early and mid-gestation.

To understand how P-gp and BCRP activity is regulated, mRNA/protein levels were assessed under the same oxygen concentrations and time-points. P-gp protein showed no differences in expression at any of the time-points investigated whereas *ABCB1* mRNA levels significantly decreased after 24- and 48-h of hypoxia in hfBECs derived in mid-gestation. BCRP protein expression was significantly downregulated after 48-h of hypoxia in early gestation hfBECs only and no differences were observed in *ABCG2* mRNA levels at any time-point or age. This disconnect between mRNA and protein is a common phenomenon that occurs for P-gp and BCRP in various tissue types, and has been reported previously [8,43,[63], [64], [65]]. Imperio et al. [43] investigated how infection mimics affect MDR transporter function and expression in an adult BEC line, hCMEC/D3. LPS (lipopolysaccharide, a bacterial infection mimic) treatment increased P-gp function, with a concomitant reduction in *ABCB1* levels, and no change in P-gp protein. Petropoulos et al. [63] assessed BCRP expression in fetal mouse brain following glucocorticoid exposure and observed a significant reduction in *Abcg2* mRNA expression, but no decrease in BCRP protein expression and an elevation in BCRP function. In the present study, protein levels were measured in whole cell lysate whereas functional assays target membrane transporter activity. Posttranslational modifications have been shown to be important in the modulation of ABC transporter function [66]. P-gp can be highly glycosylated [44] and phosphorylated. Increased phosphorylation of BCRP by Pim-1 may lead to increased protein surface translocation. This indicates that posttranslational modifications may represent a mechanism driving the changes in activity observed without changes in protein levels.

Regulation of P-gp and BCRP activity may also occur at the level of incorporation into the cell membrane. In this connection, scaffold proteins at the plasma membrane such as moesin, radixin and ezrin are involved in sustaining localization and regulating the function of transporter proteins in several organs [67,68]. Previous work in our lab has measured P-gp and BCRP protein in the cellular membrane of hfBECs derived in early and mid-gestation. P-gp in the plasma membrane was lower in hfBECs derived in mid-gestation compared to hfBECs from early gestation but there were no differences in P-gp function [8]. This suggests that lower levels of P-gp at the membrane does not necessarily equate to reduced efflux function [8]. Further, in the adult hCMEC/D3 cell line we found that membrane associated P-gp and BCRP protein levels did not correlate with their activity following exposure to infection mimics, LPS, PolyI:C and ssRNA [43]. These studies further indicate that there is no direct association between protein abundance at the cell membrane and transporter activity in BECs. However, given that membrane localization and functional modulation of P-gp and BCRP is tissue-specific [68], in future studies it would be important to investigate changes in the scaffold proteins in hfBECs following hypoxia.

The ABC transporters can also be regulated at the level of translation. miR-146a-5p can lower P-gp levels in the brain microvasculature derived from rats in status epilepticus [69], and this occurs via downregulation of the NF-κB signaling pathway [57]. Moreover, miR-331-5p, which is involved in the repression of P-gp translation, is upregulated in preterm human placentas with histological chorioamnionitis, and this is associated with a down-regulation of placental P-gp levels [70]. This indicates that specific microRNAs can regulate translation of drug transporters and may also be involved in the disconnect between levels of MDR transporter mRNA and protein which have been frequently reported. This clearly warrants further investigation.

### 4.1 Limitations of the study

Potential limitations comprise the relatively low number of independent hfBEC samples (n = 6/group) in the present study. This is due to the limited availability of human fetal brain specimens. However, previous research investigating drug transporter activity and expression in developing tissues has utilized six subjects per group in human [8,49,50,52,[70], [71], [72]] and animal [[73], [74], [75], [76]] studies. The REB requirements do not allow for clinical data collection from donors. In this connection, maternal clinical information such as age, BMI, ethnicity, parity, and fetal sex could explain, at least in part, some of the within group variability detected in some of the effects observed. Finally, our model system does not allow investigation of potential changes in blood flow, which are known to influence regulation of transporter function. Future studies incorporating flow in *in vitro* culture systems will further strengthen the interpretation of our results [77,78]. Notwithstanding, the present study is novel and shows that hypoxia modifies the activity and expression of the two primary MDR transporters in hfBECs, in an age-dependent manner.

## 5 Conclusions

In summary, we have identified specific changes in P-gp and BCRP activity and expression after short term and prolonged severe hypoxia in early and mid-gestation hfBECs. Longer-term exposure to hypoxia (48-h) significantly decreased BCRP protein and function in early gestation hfBECs. This implies that the fetal brain may become more vulnerable to exposure to potentially harmful BCRP substrates present in the maternal circulation during early development but may increase brain deposition of other endogenous substrates such as folate. In mid-gestation, severe hypoxia significantly increased P-gp and BCRP activity. Changes in P-gp function after just 6-h suggests a high sensitivity of P-gp to hypoxia in mid-gestation. Elevated activity of both transporters would potentially decrease transfer of substrates, including xenobiotics, drugs, and endogenous molecules across the fetal BBB.

## Data availability

Data associated with our study has not been deposited into a publicly available repository. All data generated or analyzed during this study are included in this article and its supplementary information files.

## Ethics approval and consent to participate

Human fetal brains were collected following elective pregnancy termination, through the Research Centre for Women's and Infants' Health BioBank based at Sinai Health System, in adherence to research ethics boards (REBs) at Sinai Health and the University of Toronto. In all cases, written informed consent (as per REB protocol number: 18-0057-E) was obtained.

## Funding

This study was funded by Canadian Institutes for 10.13039/100005622Health Research (10.13039/501100000024CIHR), Canada; grant number: FDN-148368 to 10.13039/501100000342SGM. 10.13039/501100000342SGM is also supported by a Canada Research Chair, Canada (Tier 1; CRC-2019-00386). E.B. is funded by Conselho Nacional de Desenvolvimento Científico e Tecnológico (10.13039/501100003593CNPq), Brazil; grant numbers: 10578/2020-5 and 310489/2023-7) and Fundação de Amparo à Pesquisa do Estado de Minas Gerais (10.13039/501100004901FAPEMIG), Brazil; grant number APQ-00338-18.

## CRediT authorship contribution statement

**Hafsah Mughis:** Writing – review & editing, Writing – original draft, Methodology, Investigation, Conceptualization. **Phetcharawan Lye:** Methodology, Investigation. **Guinever E. Imperio:** Methodology. **Enrrico Bloise:** Writing – review & editing, Writing – original draft, Supervision, Methodology, Funding acquisition, Conceptualization. **Stephen G. Matthews:** Writing – review & editing, Writing – original draft, Supervision, Resources, Project administration, Methodology, Funding acquisition.

## Declaration of competing interest

The authors declare that they have no known competing financial interests or personal relationships that could have appeared to influence the work reported in this paper.

## Figures and Tables

**figs1:**
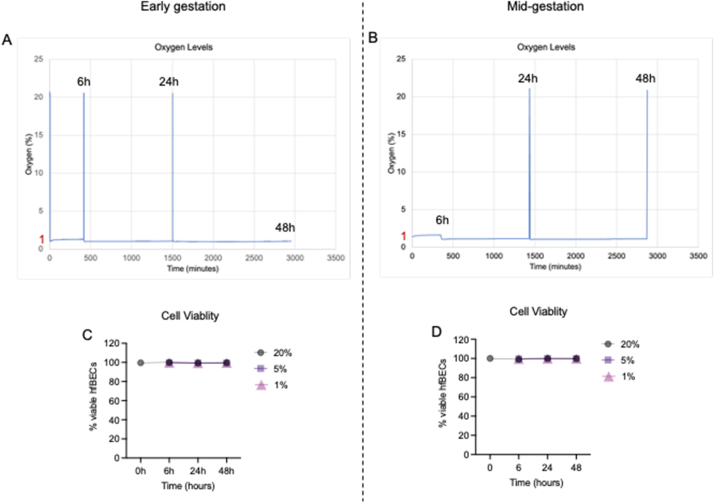


**figs2:**
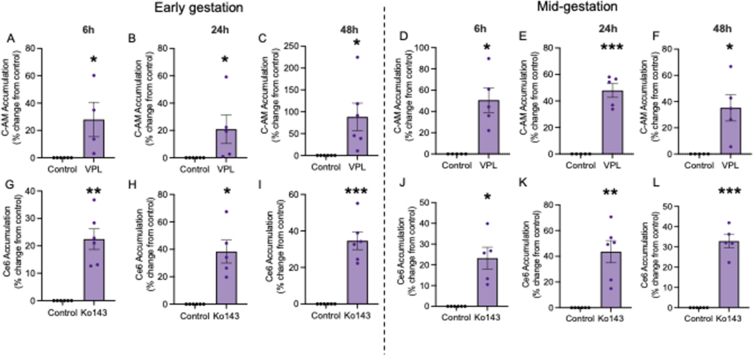


**figs3:**
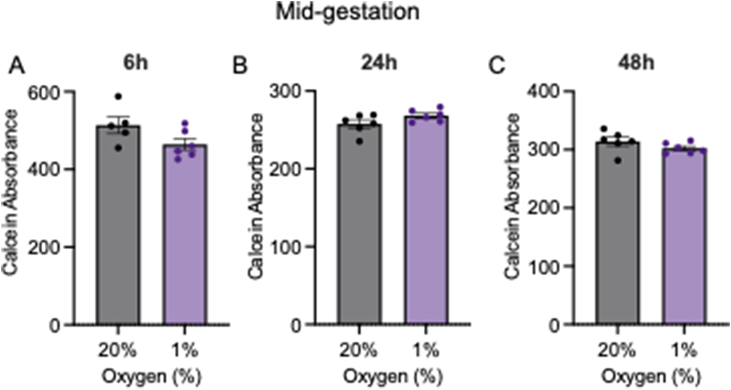


**figs4:**